# A Multicenter Matched Cohort Study of Processed Nerve Allograft and Autograft in Mixed and Motor Nerve Reconstruction

**DOI:** 10.1016/j.jhsg.2026.101146

**Published:** 2026-09-28

**Authors:** Glenn Gaston, Dominic Power, Brendan MacKay, Andrew Watt, Bauback Safa, Greg M. Buncke

**Affiliations:** ∗ OrthoCarolina, Charlotte, NC; † Queen Elizabeth University Hospital, Manchester, United Kingdom; ‡ Texas Tech University Health Sciences Center, Lubbock, TX; § The Buncke Clinic, San Francisco, CA

**Keywords:** MATCH, Nerve allograft, Nerve autograft, Peripheral nerve repair, RANGER

## Abstract

**Purpose:**

Nerve transections often require surgical intervention with a bridging material to achieve meaningful recovery (MR). We explored similar cohorts with nerve reconstruction using nerve autograft or nerve allograft. We hypothesized that motor recovery in mixed and motor nerve injuries repaired with nerve allograft is noninferior to repairs with nerve autograft.

**Methods:**

Subjects had mixed or motor nerve transections in the upper extremity distal to the axilla surgically repaired with nerve autograft or nerve allograft. Motor recovery outcomes were analyzed using the British Medical Research Council Classification (MRCC) with MR defined as ≥M3 and enhanced MR defined as ≥M4.

**Results:**

There were 24 subjects with 25 repairs in the autograft group and 23 subjects with 25 repairs in the allograft group. Motor recovery of ≥M3 was achieved in 64% and ≥M4 in 28% of autograft repairs. In contrast, motor recovery of ≥M3 was achieved in 80.0% and ≥M4 in 60% of allograft repairs. The median (interquartile range) MRCC score in the autograft cohort was 3.0 (1.0–4.0; 95% CI, 2.0–3.0) and 4.0 (3.0–4.0; 95% CI, 3.0–4.0) in the allograft cohort. The mean (SD) MRCC score in the autograft cohort was 2.5 (1.5; 95% CI, 1.9–3.1) and 3.2 (1.4, 95% CI, 2.7–3.8) in the allograft cohort. Analysis found that return of motor function in nerve allograft was as favorable as autograft.

**Conclusions:**

These findings support allograft as a reasonable alternative to autograft for upper extremity mixed and motor nerve transection injuries. Allograft performed as favorable as autograft while avoiding the need for autograft harvest and the potential donor site morbidity.

**Type of study/level of evidence:**

Therapeutic III.

Trauma, surgery, and oncological resection may result in peripheral nerve injury with loss of nerve trunk continuity and function. Clinically meaningful motor recovery is associated with some functional recovery, which can be defined as British Medical Research Council Classification (MRCC) scores ≥M3, active movement against gravity or better.^1^^,^^2^ Reconstruction of the nerve gap with bridging material, such as a nerve conduit, nerve autograft, or processed nerve allograft, is required when there is a loss of nerve tissue or when the nerve ends cannot be directly repaired without excessive tension.^3^ Historically, the only option for reconstruction of mixed and motor nerve injuries with a nerve graft has been reconstruction with nerve autograft.

The limitations of nerve gap reconstruction using autologous graft are well documented in the literature.^4^^,^^5^ Autograft nerve harvest may be associated with complications including donor nerve functional deficits, donor site pain, neuroma formation, and wound infections.^6^^,^^7^ However, a recent study reported on 43 patients who underwent sural nerve harvest, reporting no complaints related to pain, cold intolerance, itchiness, difficulties walking, or foot swelling.^8^ Although the sural nerve is commonly harvested, a clinical review of data suggested that alternatives should be considered because of the potential complications associated with its use.^6^ Both nerve conduit and nerve allograft have been successfully used in the reconstruction of peripheral nerve gaps. Clinical use of nerve allograft for digital nerve repair reported good or excellent recovery in 94% of repairs 12 months postoperative.^9^ Ducic et al^10^ found that nerve allograft returned functional improvements in 54 discrete nerve repairs, similar to outcomes of other treatments. Clinical studies have reported successful use of nerve allograft in nerve reconstruction after symptomatic neuroma excision in the lower extremity; in discontinuities up to 70 mm in the lingual and inferior alveolar nerve; and in sensory, mixed, and motor nerve reconstructions.^11^, ^12^, ^13^

A review of clinical studies found that the overall meaningful recovery of nerve autograft and nerve allograft showed no significant differences between the groups, at 71.8% meaningful sensory recovery and 56.0% meaningful motor recovery in patients repaired with nerve autografts and 81.9% meaningful sensory recovery and 58.3% meaningful motor recovery in patients repaired with nerve allografts.^14^ This study was a valuable data set when evaluating the meaningful recovery of peripheral nerves repaired with nerve bridging materials; however, the authors recognized that the study was limited by the available study designs, as many contributing studies did not have comparator groups across bridging materials.^14^ Our study aimed to provide a direct comparison of prospectively gathered data on outcomes of motor and mixed nerve gap repair using nerve autografts versus nerve allografts within a single clinical study. We hypothesized that nerve allograft motor MRCC scores would be noninferior to nerve autograft motor MRCC scores.

## Materials and Methods

### Study design

In 2008, a multicenter registry study, RANGER (Clinical Trial# NCT01526681), was created to understand efficacy and safety for the use of nerve allograft (Avance Nerve Graft, Axogen Corporation), including mixed and motor nerve repairs. In 2013, the MATCH arm was added to the registry to collect data on outcomes from nerves repaired with nerve autograft. Prospectively gathered data from the results of nerve allograft repairs in the RANGER study and nerve autograft repairs from the MATCH arm were compared in the present study with respect to their outcomes for managing nerve gaps in motor and mixed nerves.

RANGER was conducted in accordance with all applicable federal regulations, good clinical practices governing protection of human subjects, and institutional review boards. To control for potential biases and ensure RANGER and MATCH arm data robustness, all participating institutions followed their standard of care for treatment and postoperative care for the specific injury and nerve reconstruction as well as following standardized methods for chart review and data collection, which have been previously described for the RANGER registry.^13^^,^^15^ Management of the resultant nerve gap using nerve autograft or nerve allograft was performed at the discretion of the treating surgeon following discussion with the patient. All data were deidentified and entered into the centralized RANGER database.

The RANGER database was queried for subjects with a mixed or motor nerve transection injury of the upper extremity (distal to the axilla) that was surgically repaired using nerve autograft (MATCH arm, from initiation in January 2013 to September 2022) or nerve allograft (RANGER, from initiation in November 2008 to September 2022). This query resulted in 128 nerve autograft repairs and 564 nerve allograft repairs (Fig. 1). Repairs were included if they underwent tension free end-to-end nerve coaptation on both the proximal and distal portion of the nerve reconstruction within 7 months (using the average of 30.4 days per month this is rounded up to 213 days) of injury, completed follow-up assessments within a suitable time frame to determine the outcome of the repair based on injury location, and complied with site-specific postoperative care procedures and assessments to determine the outcome of the repair. Repairs for nerve gaps >70 mm (as nerve allograft is only available in lengths up to 70 mm), amputation, or avulsion mechanisms of injury; reconstructions that may be confounded by nerve or tendon transfers; repairs with postoperative time >213 days; nerve repairs with an unknown level of injury; and repairs with an absence of outcomes data and insufficient outcomes or follow-up or unknown level of injury/repair were excluded from analysis. This process served to reduce the heterogenicity of the data, decrease variability from potential outliers, and enhance meaningful comparisons from similar samples between the cohorts. This query resulted in 25 nerve autograft and 25 nerve allograft repairs.

**Figure 1:**
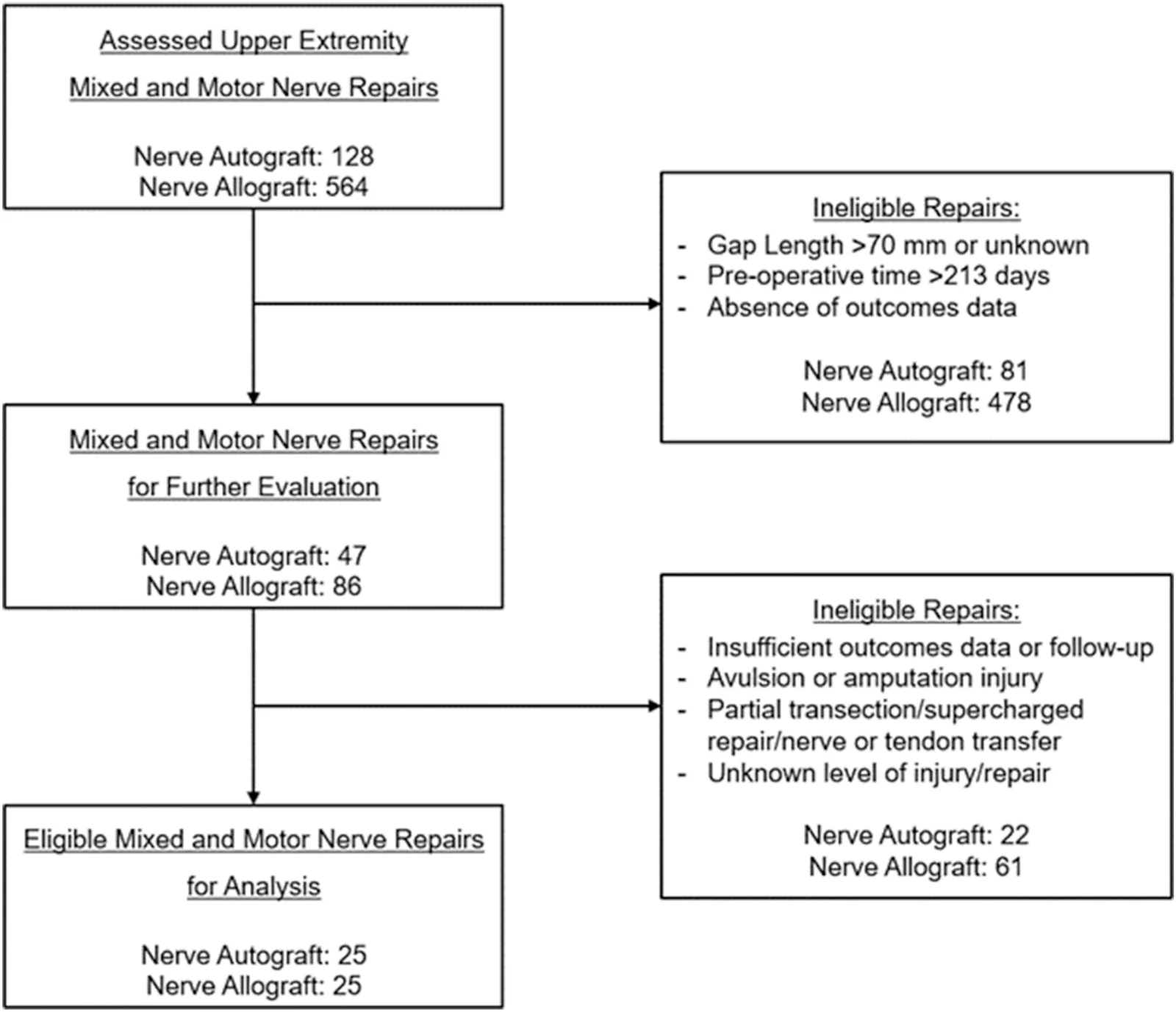
CONSORT diagram for mixed and motor nerve repairs assessed for analysis.

Functional outcomes were collected from standardized forms and nerve-specific functional outcome measures (eg, wrist and finger extension for radial nerve repairs, or thumb abduction for median nerve repairs). MRCC scores of motor recovery were used for outcomes assessments to align nerve-specific outcome measures across injuries, sites, and study arms (Table 1). We selected MRCC scoring for clinical assessments because of its long history of clinical use, high adoption in the literature, suitability to evaluate peripheral nerve recovery across various anatomical locations, and substantial inter-rater and intra-rater reliability.^16^^,^^17^ For those participants and repairs meeting inclusion, the authors reviewed functional outcome measures to convert the nerve-specific outcomes to MRCC scores.

**Table 1:** Medical Research Council Classification of Motor Recovery

Score	Definition
M0	No contraction
M1	Flicker or trace of contraction
M2	Full range of active movement, with gravity eliminated
M3	Active movement against gravity
M4	Active movement against gravity and resistance
M4+	Strong movement against resistance
M5	Normal/full power

### Clinical evaluation

Peripheral nerve repair was performed with nerve autograft or nerve allograft as a standard-of-care (ie, not randomized). Subjects completed a minimum evaluable postoperative visit, which was based on a regeneration rate of 1 mm/day to accommodate for the distance from injury to the target tissue to allow for sufficient time to achieve meaningful recovery: 180 days for repairs in the hand, 270 days for repairs in the wrist, 365 days for repairs in the forearm and elbow, and 540 days for repairs above the elbow.^18^ MRCC score for a distal target muscle was used for analysis, with the most distal target muscle used for nerves with multiple reinnervation targets.

Data were collected from clinical site patient medical records including subject’s medical history and demographics, nerve injury, concomitant injuries, nerve repair/implant, history of prior surgical treatments, preoperative assessments, and postoperative follow-up assessments. Demographic and baseline characteristics included age, sex reported in the medical record, mechanism of injury, gap length, number of nerves repaired, and number of concomitant injuries, which were summarized descriptively by treatment type. Functional recovery assessments were performed by qualified evaluators, which included treating and nontreating surgeons, therapists, and trained research personnel. These functional recovery assessments were recorded as muscle function (ie, strength and active range of motion) or MRCC of motor recovery scores. All MRCC scores were reviewed and verified by the treating surgeons and authors. Efficacy endpoints included the distribution of motor recovery in mixed and motor nerve repairs (M0 through M5 on MRCC of motor recovery scale) and proportion of subjects with meaningful motor recovery in mixed and motor nerve repairs (defined as M3 or better on MRCC of motor recovery scale).

### Data analysis

Descriptive statistics were used to summarize the outcomes data for mixed and motor nerve repairs, with comparisons between nerve autograft and nerve allograft repairs. Unless stated otherwise, the term “descriptive statistics” refers to the number of subjects or repairs, mean, median, SD, 95% CI, interquartile range (IQR), minimum, and maximum for continuous data and frequencies and percentages for categorical data. Demographic, follow-up, and nerve injury variables were tested for significant differences using two-sample *t* tests and Fisher exact tests. The term “treatment cohort” refers to the treatment received from RANGER (nerve allograft) or MATCH (nerve autograft). Data in RANGER and MATCH were reported by nerve repair. Given the nature of injuries in RANGER and MATCH, it was possible for an individual to contribute more than one nerve repair to the database; however, subjects contributed to only one treatment cohort.

A histogram was generated to display the MRCC distribution of motor recovery for each treatment cohort. The noninferiority of nerve allograft to nerve autograft for MRCC functional motor recovery was evaluated by comparing the distribution of MRCC scores between cohorts. MRCC is an ordinal scale in which each integer point represents a clinically meaningful functional change; therefore, we prespecified a noninferiority margin of 1 point. Let Δ denote the difference in median MRCC score (nerve allograft − nerve autograft). The noninferiority hypothesis was H_0_: Δ ≤ −1.0 (allograft inferior by more than the margin) vs H_a_: Δ > −1.0 (allograft noninferior). A one-sided Wilcoxon rank-sum (Mann–Whitney) test was used to compare MRCC score distributions between cohorts. The difference in MRCC score location (Δ) was estimated using the Hodges-Lehmann method, and a one-sided 95% confidence interval for Δ was used to assess noninferiority. Noninferiority was concluded if the lower bound of this interval excluded −1.0, which is equivalent to obtaining a one-sided *p* value ≤ α for the above noninferiority hypothesis. A priori sample size calculations indicated that 33 repairs per treatment cohort (total n = 66) were required to demonstrate noninferiority of nerve allograft to nerve autograft on the MRCC motor score with 85% power, assuming a one-sided α = 0.05 and a noninferiority margin of 1 MRCC grade, using a one-sided Wilcoxon rank-sum (Mann–Whitney) test. These calculations were based on the assumptions of similar MRCC score distributions, with no difference in medians between cohorts. The proportions of meaningful motor recovery in mixed and motor nerve repairs were summarized by cohort, with meaningful recovery (MR) evaluated at ≥M3 and enhanced MR at ≥M4. A Spearman correlation was performed to evaluate MRCC scores for preoperative interval, follow-up days, gap length, number of concomitant injuries, and age.

## Results

### Subject and injury demographics

Forty-seven nerve autograft repairs and 86 nerve allograft repairs were identified in the database. Subjects were excluded for insufficient outcomes, unknown injury level, supplemental nerve or tendon transfers (supercharged), or amputation and avulsion injuries (Fig. 1). Data analysis included 25 nerve autograft repairs in 24 subjects and 25 nerve allograft repairs in 23 subjects. Twenty-nine surgeons from 13 sites contributed to study data, including 12 surgeons from 7 sites for nerve autograft and 18 surgeons from 8 sites for nerve allograft. All subjects underwent surgical repair within 207 days of injury.

Demographics statistical testing revealed that mean (SD) age of nerve autograft was 40.4 (16.7) years, which was significantly higher than mean age of nerve allograft at 29.4 (13.5) years (*P* < .05, Table 2). The follow-up time, gap length, number of concomitant injuries, mechanism of injury, location of injury, and nerve repaired showed no statistical differences between cohorts (Tables 3 and 4). The mean (SD) gap length was 42.7 (15.9) mm for nerve autograft and 34.1 (17.0) mm and nerve allograft. The most common mechanism of injury for both cohorts was sharp laceration (32.0% and 48.0% for nerve autograft and nerve allograft, respectively), and the most common location was the forearm (40.0% and 36.0% for nerve autograft and nerve allograft, respectively). Moreover, most subjects had at least one concomitant injury. The most injured nerves for both cohorts were median, ulnar, and radial, in that order. The mean (SD) preoperative interval was significantly higher in the nerve autograft cohort at 55.5 (65.6) days than the allograft cohort at 16.1 (37.4) days (*P* < .05). There was no correlation between MRCC scores and age, follow-up time, preoperative interval, gap length, and number of concomitant injuries.

**Table 2:** Demographics

Demographics Category	Nerve Autograft	Nerve Allograft
Age at Enrollment		
N	24	23
Mean (SD)	40.3 (16.7)∗	29.4 (13.5)∗
Median	35.0	27.0
Min, Max	14.0, 70.0	16.0, 64.0
Sex		
Male	21 (87.5%)	20 (87.0%)
Female	3 (12.5%)	3 (13.0%)
Race		
American Indian or Alaska Native	0	0
Asian	3 (12.5%)	1 (4.3%)
Black or African American	4 (16.7%)	1 (4.3%)
Native Hawaiian or Other Pacific Islander	0	0
White	16 (66.7%)	5 (21.7%)
Unknown	1 (4.2%)	16 (69.6%)
Ethnicity		
Not Hispanic or Latino	22 (91.7%)	6 (26.1%)
Hispanic or Latino	1 (4.2%)	3 (13.0%)
Unknown	1 (4.2%)	14 (60.9%)
Number of Nerves Repaired		
1	23 (95.8%)	21 (91.3%)
2	1 (4.2%)	2 (8.7%)

N, number of subjects.

∗ *P* < .05.

**Table 3:** Follow-up Information

Follow-up (d)	Nerve Autograft	Nerve Allograft
n	25	25
Mean (SD)	855 (759.5)	907 (690.0)
Median	552	691
Min, Max	270, 3684	186, 3089

Note: One nerve autograft subject contributed two repairs. Two nerve allograft subjects contributed two repairs. Follow-up is the number of days from Day 0 to the date of study completion or last recorded assessment in the database.

n, number of repairs.

**Table 4:** Nerve Injury Variables

Nerve Injury Variable	Nerve Autograft (N = 24)	Nerve Allograft (N = 23)
Preoperative Interval (d)		
n	25	25
Mean (SD)	55.5 (65.6)∗	16.1 (37.4)∗
Median	23	1.0
Min, Max	0, 207	0, 171
Gap Length (mm)		
n	25	25
Mean (SD)	42.7 (15.9)	34.1 (17.0)
Median	40.0	30.0
Min, Max	15.0, 70.0	13.0, 70.0
Number of Concomitant Injuries		
n	25	25
Mean (SD)	1.4 (1.1)	1.7 (0.9)
Median	2	2
Min, Max	0, 3	0, 3
Mechanism of Injury		
Sharp laceration	8 (32.0%)	12 (48.0%)
Gunshot/blast	6 (24.0%)	3 (12.0%)
Saw laceration	4 (16.0%)	4 (16.0%)
Crush/compression	2 (8.0%)	3 (12.0%)
Iatrogenic	3 (12.0%)	1 (4.0%)
Blunt laceration	1 (4.0%)	0
Fall	1 (4.0%)	0
MVC	0	1 (4.0%)
Other	0	1 (4.0%)
Location of Injury – Upper Extremity		
Forearm	10 (40.0%)	9 (36.0%)
Upper arm	5 (20.0%)	3 (12.0%)
Elbow	5 (20.0%)	3 (12.0%)
Wrist region	4 (16.0%)	4 (16.0%)
Digital/hand	1 (4.0%)	5 (20.0%)
Unspecified	0	1 (4.0%)
Nerve Repaired – Mixed		
Median nerve	8 (32.0%)	14 (56.0%)
Ulnar nerve	8 (32.0%)	7 (28.0%)
Radial nerve	8 (32.0%)	3 (12.0%)
Musculocutaneous nerve	1 (4.0%)	1 (4.0%)

Note: One nerve autograft subject contributed two repairs. Two nerve allograft subjects contributed two repairs.

MVC, motor vehicle collision; N, number of subjects; n, number of repairs.

∗ *P* < .05.

### Efficacy assessments

A majority of nerve autograft subjects (64.0%, 95% CI 42.5–82.0%) achieved motor MR (≥M3) compared with a higher proportion in the nerve allograft group (80.0%, 95% CI, 59.3–93.2%). Enhanced MR (≥M4) was observed in 28% (95% CI, 12.2–48.9%) of autograft subjects and 60% (95% CI, 38.7–78.9%) of allograft subjects (Figs. 2 and 3, Table 5). The observed median (IQR) MRCC score in the autograft cohort was 3.0 (1.0–4.0; 95% CI, 2.0–3.0) and 4.0 (3.0–4.0; 95% CI, 3.0–4.0) in the allograft cohort, yielding a median between group difference of 1.0 MRCC unit. The observed mean (SD) MRCC score in the autograft cohort was 2.5 (1.5, 95% CI, 1.9–3.1) and 3.2 (1.4, 95% CI, 2.7–3.8) in the allograft cohort, with a mean difference of 0.7 MRCC units (95% CI –0.1–1.6). Observed MRCC outcomes were more favorable than anticipated in the a priori analysis, with a greater separation between cohorts because of a shift in higher MRCC scores in the nerve allograft group (Fig. 4, Table 6).

**Figure 2:**
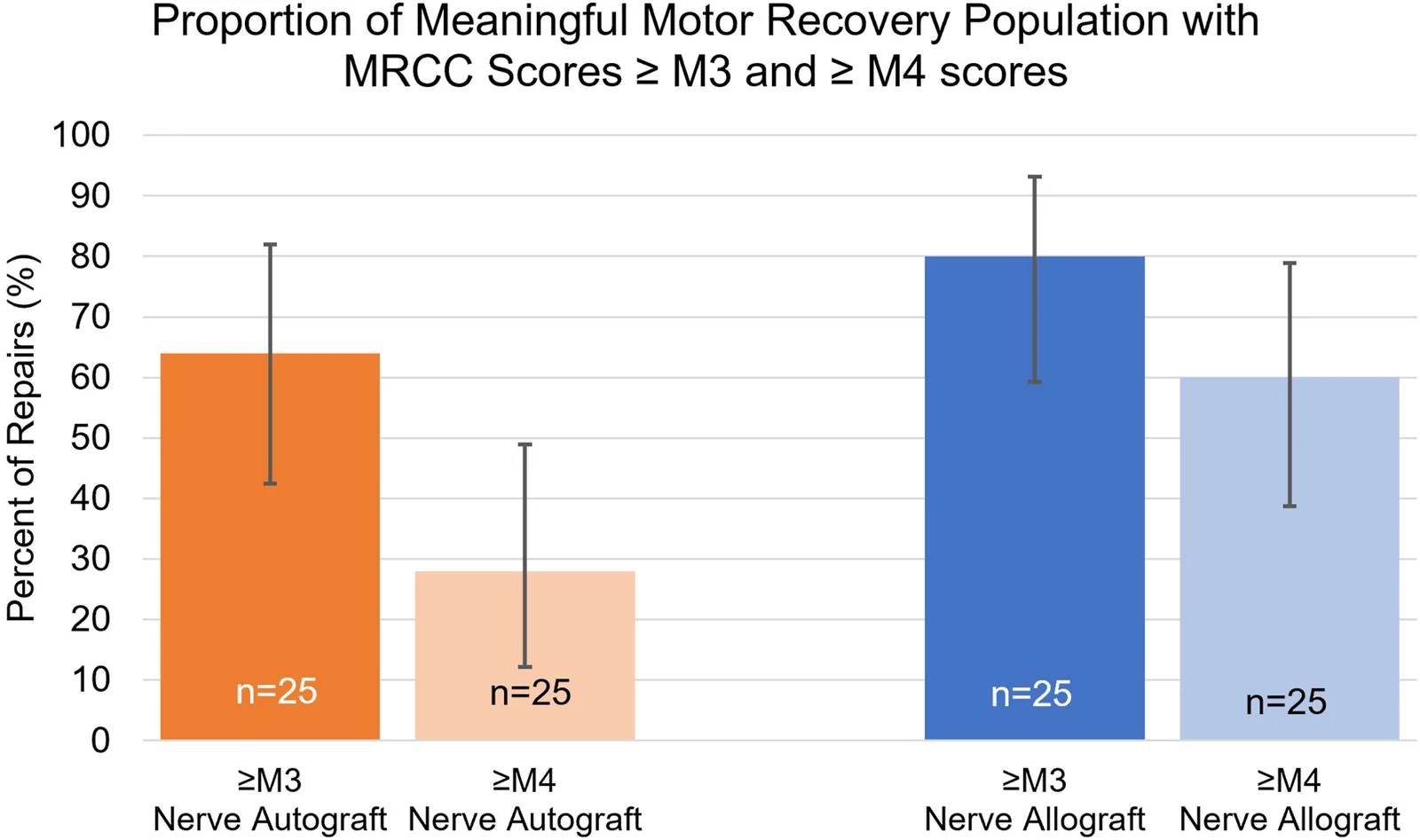
Proportion of repairs achieving motor recovery, with MRCC scores delineated at meaningful recovery ≥M3 and enhanced meaningful recovery≥M4. Data presented as proportion and 95% CI.

**Figure 3:**
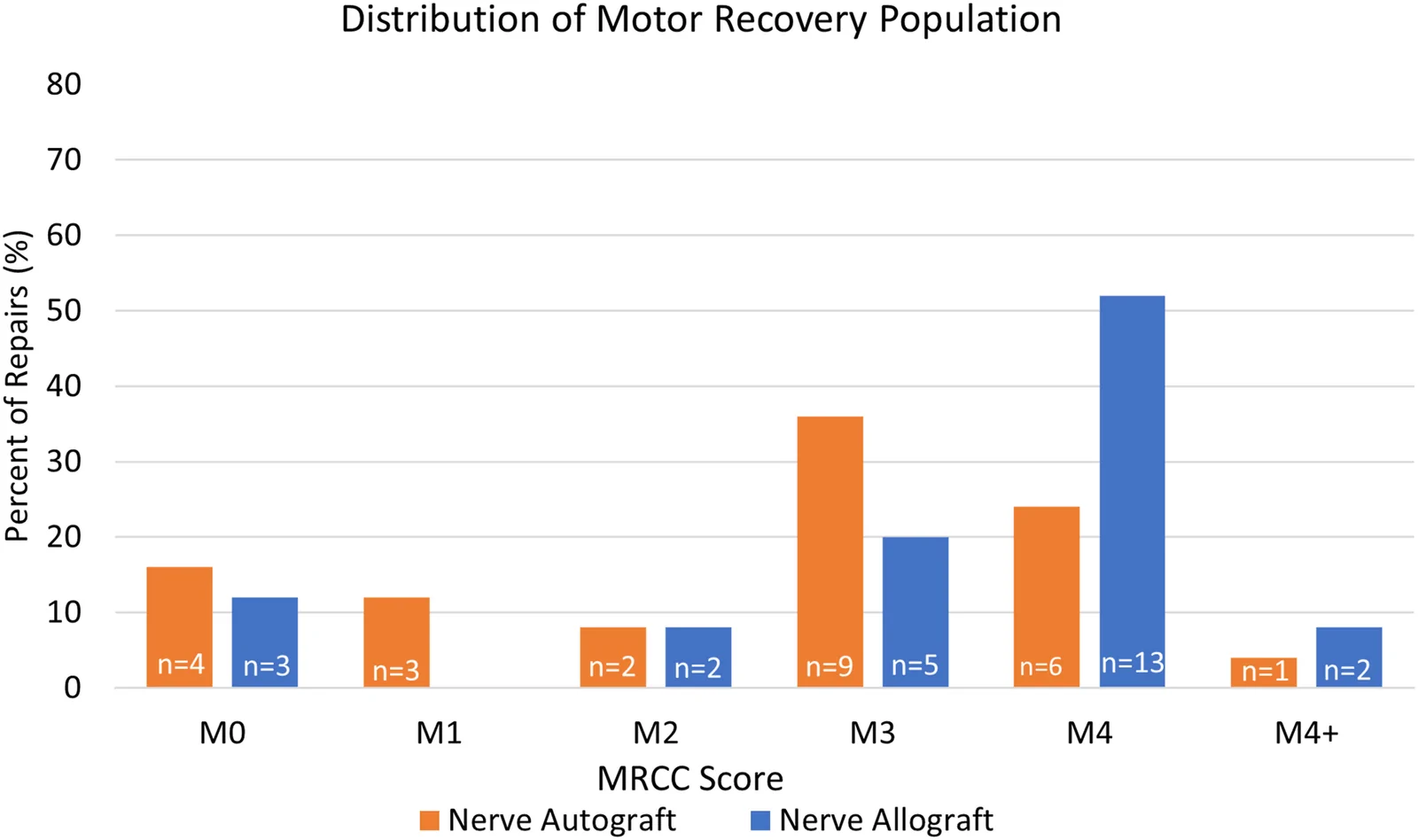
Distribution of MRCC motor recovery.

**Table 5:** MRCC Outcomes

MRCC Outcomes	Nerve Autograft (n =25)	Nerve Allograft (n = 25)
MRCC		
M0	4 (16.0%)	3 (12.0%)
M1	3 (12.0%)	0 (0.0%)
M2	2 (8.0%)	2 (8.0%)
M3	9 (36.0%)	5 (20.0%)
M4	6 (24.0%)	13 (52.0%)
M4+	1 (4.0%)	2 (8.0%)
Meaningful Motor Recovery∗		
Number repairs achieving meaningful motor recovery	16	20
Proportion (95% CI) of ≥M3 recovery†	64.0% (42.5%–82.0%)	80.0% (59.3%–93.2%)
Proportion (95% CI) of ≥M4 recovery†	28.0% (12.2%–48.9%)	60.0% (38.7%–78.9%)

Note: If a subject received more than one repair, each repair was treated as an independent data point.

n, number of repairs in treatment group.

∗ Meaningful recovery is evaluated at MRCC score ≥M3 and ≥M4.

† 95% CIs are determined using the normal approximation.

**Figure 4:**
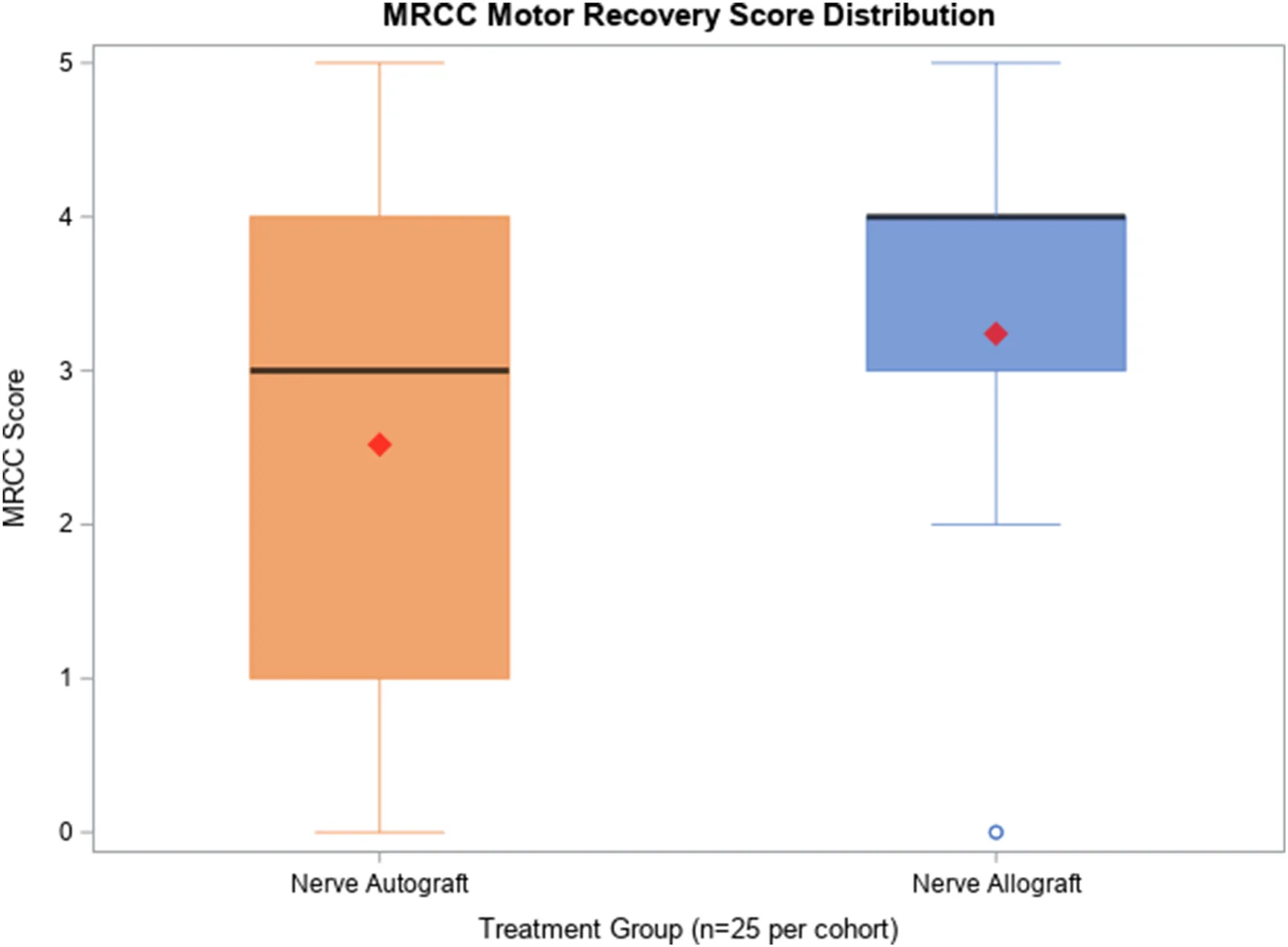
MRCC Motor Recovery Score distribution.

**Table 6:** MRCC Motor Recovery Outcomes and Noninferiority Analysis

MRCC	Nerve Autograft (n = 25)	Nerve Allograft (n = 25)
Mean (SD)	2.5 (1.5)	3.2 (1.4)
Mean 95% CI∗	1.9–3.1	2.7–3.8
Median (IQR)	3.0 (1.0–4.0)	4.0 (3.0–4.0)
Median 95% CI†	2.0–3.0	3.0–4.0
Between Group Comparisons		
Mean difference (95% CI)	--	0.72 (-0.11–1.55)
Hodges–Lehmann location shift (95% CI)	--	1.0 (0.0–1.0)
Noninferiority *P* value‡	--	<.0001

∗ 95% CIs for the mean were calculated using the t distribution.

† 95% CIs for the median were calculated using a distribution-free method based on order statistics.

‡ One-sided Wilcoxon rank-sum noninferiority test (NI margin = −1.0 MRCC grade) using Hodges–Lehmann location shift estimation.

Noninferiority of nerve allograft relative to autograft was demonstrated, as the lower bound of the one-sided 95% CI for the estimated location shift for the Hodges–Lehmann MRCC location shift exceeded the prespecified margin of −1.0 MRCC grade. The null hypothesis of inferiority was rejected (*P* < .05), and conclusions were based on confidence intervals and hypothesis testing rather than observed power. Although enrollment was below the a priori target sample size, the magnitude and precision of the observed treatment effect supported robust conclusions regarding noninferiority of nerve allograft repairs compared to nerve autograft repairs.

## Discussion

Surgical advances in nerve gap reconstruction for mixed and motor nerves has been limited despite well-established use of intercalary nerve grafts to reconstruct nerve gaps.^10^ A recent study found comparable meaningful recovery rates between nerve autograft and allograft, independent of nerve gap size.^14^ Although outcome measures vary across clinical studies, our study used a single outcome measure, MRCC score, to evaluate the distribution of motor recovery after nerve repair with nerve autograft and nerve allograft in mixed and motor nerves. MRCC scores were used to remove subjective bias from reporting and allow for potential future comparisons study comparisons.

Meaningful functional recovery was observed in a majority of the subjects in both cohorts after repair of mixed and motor peripheral nerve injuries. The distribution of MRCC scores for nerve allograft was noninferior to nerve autograft, as the lower bound of the median difference 95% CI was 0, which was above the noninferiority margin difference of –1.0. The nerve allograft cohort exhibited a 16% higher rate of ≥M3 recovery than in autograft (80.0% vs 64.0%) and a 32% higher rate of ≥M4 recovery in allograft than in autograft (60.0% vs 28.0%). Further, the allograft cohort showed a mean MRCC score of 3.2, which was 0.7 units higher than in the nerve autograft cohort (mean MRCC score 2.5). These findings suggest that allograft performance was as favorable as autograft performance. Although this cohort study was not randomized or blinded, this functional recovery bolsters the evidence of effective reconstruction of mixed and motor nerves with allograft and mirrors the results of the study by Lans et al.^14^

There were limited statistical differences between cohort demographics. On average, nerve autograft subjects (40.3 ± 16.7 years) were older than nerve allograft subjects (29.4 ± 13.5 years, *P* < .05), which is of unknown clinical significance. Notably, Mackay et al^19^ reported satisfactory motor recovery in 60% to 67% of subjects ≤40 years old, which dropped to 47.6% in subjects >40 years of age. The mean and median age of both study populations was ≤40 years, suggesting that age did not likely contribute to meaningful recovery differences. The mean preoperative interval was longer in the nerve autograft cohort than in nerve allograft (mean: 55.5 days vs 16.1 days, *P* < .05); however, the difference in median preoperative interval was only a few weeks (23 days vs 1 day) and is not a clinically meaningful difference. Although immediate repair is ideal and delays beyond 6 months postinjury are associated with diminished functional motor recovery, the decline is gradual with irreversible motor endplate degradation occurring up to 1 year after nerve transection.^19^ Further, there was no correlation between MRCC scores and age, follow-up time, preoperative interval, gap length, and number of concomitant injuries.

The RANGER study represents the largest ongoing, multisite collection of peripheral nerve repair data of its kind. Nonetheless, there are limitations of RANGER and MATCH, including the nonrandomized, open-label design; however, the nature of peripheral nerve repair does not permit blinding. Although the site evaluators and patients were not blinded to treatment, MRCC scores are quantitative data that report an objective level of recovery based on physical performance. In addition, this registry collects standard of care outcomes appropriate to nerve injury, and thus the sample sizes for outcome measures vary according to the availability of such data with sufficient follow-up. Surgeons contributed to on average 1.6 subjects each, which provides a representative surgeon population, but may decrease the inter-rater reliability because of the large number of evaluators and surgeons contributing subjects to the study.

Transection of a mixed nerve or motor nerve results in total loss of function in the target muscle. Surgical repair of the injured nerve with nerve allograft demonstrated a meaningful recovery rate of 80.0% compared to a rate of 64.0% for repairs with nerve autograft. The distribution of motor recovery in the nerve allograft cohort was noninferior to the nerve autograft cohort. The totality of the real-world data contained in the RANGER registry and MATCH arm provides useful insights into the utilization, functional recovery, and safety that can be expected in routine clinical practice. Despite the study limitations, the data collected through the RANGER registry and MATCH arm offers information crucial to the surgical decision-making process and this study adds to the available efficacy data of allograft in returning motor function after nerve reconstruction.

## Ethics declaration

Written informed consent to take part in the study and to publish the article has been obtained from all participants or their legal representatives. The privacy rights of participants have been observed.

This study included organ or tissue donors. This study includes human biological material and consent was obtained by donors, or their next of kin or legal representatives, for use in this study and for publication of the article. The samples used in this research were not sourced from executed prisoners or prisoners of conscience.

This study was performed in compliance with relevant laws, regulatory frameworks and guidelines where the research took place. This study was conducted in accordance with CONSORT. Ethics committee approval was not required under relevant laws and institutional guidelines. RANGER was conducted in accordance with all applicable federal regulations, good clinical practices governing protection of human subjects, and institutional review boards.

The results of this clinical trial and any associated work have been posted in a registry. This clinical trial was registered with number Clinical Trial# NCT01526681 (RANGER).

## Conflicts of Interest

The authors are consultants for various companies including: Dr Gaston (Checkpoint Surgical), Dr Safa (Axogen, Biocircuit Technologies), Dr MacKay (Axogen), Dr Power (Tissium, 4D Medical), and Dr Buncke – N/A (competing interest). No benefits in any form have been received or will be received related directly to this article.
